# *Lithothamnion muelleri* Treatment Ameliorates Inflammatory and Hypernociceptive Responses in Antigen-Induced Arthritis in Mice

**DOI:** 10.1371/journal.pone.0118356

**Published:** 2015-03-20

**Authors:** Vivian V. Costa, Flavio A. Amaral, Fernanda M. Coelho, Celso M. Queiroz-Junior, Bruna G. Malagoli, Jose Hugo S. Gomes, Fernando Lopes, Kátia D. Silveira, Daniela Sachs, Caio T. Fagundes, Lívia D. Tavares, Vanessa Pinho, Tarcilia A. Silva, Mauro M. Teixeira, Fernão C. Braga, Danielle G. Souza

**Affiliations:** 1 Laboratório de Interação Microrganismo-Hospedeiro, Departamento de Microbiologia, ICB, UFMG, Belo Horizonte, MG, Brazil; 2 Imunofarmacologia, Departamento de Bioquímica e Imunologia, ICB, UFMG, Belo Horizonte, MG, Brazil; 3 Interdisciplinary Research Group in Infectious Diseases, Singapore-MIT Alliance for Research and Technology, Singapore, Singapore; 4 Departamento de Clínica, Patologia e Cirurgia Odontológicas, Faculdade de Odontologia, UFMG, Belo Horizonte, MG, Brazil; 5 Departamento de Produtos Farmacêuticos, Faculdade de Farmácia, UFMG, Belo Horizonte, MG, Brazil; 6 Departamento de Morfologia, ICB, UFMG, Belo Horizonte, MG, Brazil; 7 Departamento de Física e Química, Instituto de Ciências Exatas da Universidade Federal de Itajubá (UNIFEI), Itajubá, MG, Brazil; Queen Mary University of London, UNITED KINGDOM

## Abstract

Rheumatoid Arthritis (RA) is a chronic disease characterized by persistent inflammation and pain. Alternative therapies to reduce these symptoms are needed. Marine algae are valuable sources of diverse bioactive compounds. *Lithothamnion muelleri* (Hapalidiaceae) is a marine algae with anti-inflammatory, antitumor, and immunomodulatory properties. Here, we investigated the potential anti-inflammatory and analgesic activities of *L*. *muelleri* in a murine model of antigen-induced arthritis (AIA) in mice. Our results demonstrate that treatment with *L*. *muelleri* prevented inflammation and hypernociception in arthritic mice. Mechanistically, the crude extract and the polysaccharide-rich fractions of *L*. *muelleri* may act impairing the production of the chemokines CXCL1 and CXCL2, and consequently inhibit neutrophil influx to the knee joint by dampening the adhesion step of leukocyte recruitment in the knee microvessels. Altogether our results suggest that treatment with *L*.*muelleri* has a potential therapeutic application in arthritis treatment.

## Introduction

Rheumatoid arthritis (RA) is one of the leading chronic inflammatory disorders in humans, affecting around 1% of the industrialized world population. The inflammatory response of the joint synovial membrane and periarticular tissues is conveyed by influx and activation of immune cells and release of inflammatory mediators (such as chemokines and cytokines), resulting in tissue destruction and dysfunction, leading to significant morbidity and mortality **[1–2].** Neutrophils are abundant in the joints of RA patients during the acute episodes of the disease **[3–5],** and these cells are important to contribute to the local production of inflammatory mediators and to inflict joint damage, perpetuating the inflammatory process **[5–8].** The treatment of RA rests on 2 main approaches: treating symptoms with non-steroidal anti-inflammatory drugs (NSAIDs) and modifying disease progress with disease-modifying antirheumatic drugs (DMARDs) **[9].** These therapies, specially recently introduced biological DMARDs such as antibodies and other inhibitors of cytokines **[10–11],** have a high cost, need parental routes, and show variable response rates among patients. In addition, they still present undesirable side effects that impact negatively on patient’s quality of life **[9].** Thus, it is not uncommon that RA patients seek alternative therapies concomitantly to trying conventional medications.

The use of alternative therapies—group of practices or products that are not part of conventional medicine (NCCAM, 2011) **[12]** is prevalent during chronic painful conditions, such as RA and osteoarthritis. In fact, 60–90% of rheumatology patients make use of some kind of alternative medicine **[13–14].** Nevertheless, the regulatory status of these products in some countries allows their commercialization without previous demonstration of efficacy and safety **[12]**. This is the case of some marine algae derived products, such as those from *Lithothamnion* spp., which are currently marked as dietary supplements in different countries (e.g. Vitality 50^+^ in Brazil and Aquamin F in USA) to treat numerous disorders, including osteoarthritis (OA). However, in most cases their efficacy is subjective and empirical, thus requiring scientific investigation of their alleged properties to validate their use **[15–16].**

*Lithothamnion muelleri* Lenormand ex Rosanoff (Hapalidiaceae) is a marine red alga characterized by high contents of minerals, especially calcium and magnesium carbonates, which occur as calcite crystals in the cell walls. Species of *Lithothamnion* are sources of potentially bioactive sulfated polysaccharides **[17–18]** and some of them elicited anti-inflammatory response in a model of skin inflammation in humans **[19]** Moreover, mineral-rich extracts obtained from *Lithothamnion calcareum* have been shown to reduce the inflammation associated to gastrointestinal polyps **[20],** whereas the treatment of patients diagnosed with moderate to severe osteoarthritis with *Lithotaminium* sp. (Aquamin F) provided relief of knee OA symptoms in two randomized controlled pilot studies **[15–16].** Recently, we reported the chemical characterization of polysaccharide-rich fractions from *Lithothamnion muelleri* and demonstrated their cell antiadhesive properties after lipopolysaccharide (LPS) administration *in vivo* **[18]**, as well its antiherpes activity *in vitro* **[21].** We also demonstrated that *L*. *muelleri* controls inflammatory responses, tissue injury and lethality associated with Graft-*versus*-Host disease in mice **[22].** Despite these evidences, the effect of *L*. *muelleri* in other experimental inflammatory conditions remains to be evaluated. Therefore, the aim of the current study was to investigate the potential anti-inflammatory and anti-hypernociceptive effects of a dietary supplement based on *L*. *muelleri* in an experimental model of antigen-induced arthritis (AIA) in mice.

## Materials and Methods

### Mice

This study was carried out in strict accordance with the Brazilian Government's ethical and animal experiments regulations (Law 11794/2008). Animal care and handling procedures were in accordance with the guidelines of the International Association for Study of Pain—IASP **[23]**. The experimental protocol was approved by the Committee on the Ethics of Animal Experiments of the Universidade Federal de Minas Gerais (CETEA/UFMG, Permit Protocol Number 165/2009). Eight-week-old male C57BL/6j mice were obtained from the Centro de Bioterismo of the Universidade Federal de Minas Gerais (UFMG, Brazil) and maintained in the animal facilities of the Department of Microbiology, Instituto de Ciências Biológicas (UFMG). Mice were housed under standard conditions (temperature and humidity) and had free access to commercial chow and filtered water.

### Antigen-induced arthritis (AIA)

The experimental model of antigen-induced arthritis (AIA) in mice was performed as described earlier **[6].** Briefly, C57BL/6j mice were immunized on day 0 with intradermal injection of 500 μg mBSA (Methylated Bovine Serum Albumin, Sigma-Aldrich, Saint Louis, MO, USA) in 50 μL phosphate buffered saline (PBS) emulsified in 50 μL Freund’s complete adjuvant (CFA; Sigma-Aldrich). Fourteen days later, the antigen challenge was performed by injecting 10 μg mBSA (in 10 μL sterile saline) in the right knee joint of each mouse. All procedures were performed under ketamine/xylazine anesthesia and all efforts were made to minimize animal suffering. Mice were monitored every 12 hours. In all experiments, mice were euthanized 24h later for evaluation. Euthanasia was performed under overdose of ketamine/xylazine anesthesia followed by cervical dislocation. Non-immunized mice and immunized mice joint-challenged with PBS were considered as negative controls.

### Therapies

Extracts of the marine algae *L*. *muelleri* are currently marketed by Phosther Algamar LTDA (Brazil) as a dietary supplement (Vitality 50+). The product is registered and approved by the Brazilian National Health Vigilance Agency (number 25003.040502/97 6.2119.0001.001-1). The crude algal material (in this study denominated as *L*. *muelleri)* was initially donated as a whitish granulate named marine mineral concentrate. According to the company, the alga was washed sequentially with tap water and distilled water to remove salt and all visible epiphytes. In the sequence, it was ground in a ball mill and dried in a ventilated oven to afford the granulate. The species was identified by Dr. Maria Carolina M. de O. Henriques, Instituto Biodiversidade Marinha, Rio de Janeiro, Brazil.

A polysaccharide-rich fraction from *L*. *muelleri* (here described as FR), whose detailed chemical composition has been recently described **[21],** was also investigated. FR was obtained by extraction of the algal material with 1% (w/v) Na_2_CO_3_ aqueous solution at 60°C, for 2 h, under mechanical stirring, followed by precipitation of the polysaccharides with ethanol and dialysis against water through a cellulose membrane. FR contains about 30% carbohydrates, 12% sulfates, 3.4% proteins and 5% uronic acids, with an average molecular weight of 46 KDa. Galactose (34%) and glucose (20%) are the major carbohydrates found in FR, followed by mannose (15%), xylose (14%), rahmnose (13%) and arabinose (4%). In parallel, the effects of calcium carbonate (CaCO_3_), a major constituent of the crude algal material was evaluated.

Mice were treated (oral gavage) twice a day (12/12 hours) with *L*. *muelleri* (10, 30 or 100 mg.kg^−1^, in carboxymethylcellulose [CMC] 0.5% in filtered water), CaCO_3_ (100 mg.kg^−1^, dissolved in CMC 0.5% in filtered water) or FR (1 mg.kg^−1^, dissolved CMC 0.5% in filtered water) for 10 days, from day 4 after immunization until the end of the experiment (Day 14). In the time-response experiments, mice were also treated with *L*. *muelleri* (100 mg.kg^−1^, in CMC 0.5% in filtered water) during 5 days before the end of the experiments (Day 10 to day 14 after immunization). The control group (Vehicle) comprised immunized and joint-challenged mice treated (for 10 days, twice a day, by oral gavage) with CMC 0.5% in filtered water.

### Knee joint evaluation

Twenty-four hours after antigen challenge the knee cavity of the mice (n = 7 mice/group) was washed with PBS (2 x 5 μl) for counting of total (Neubauer chamber) and differential leucocytes (cytospin preparations [Shandon III; Thermo Shandon, Frankfurt, Germany] stained with May-Grünwald-Giemsa stain). After PBS wash, the periarticular tissue was removed from the joint for evaluation of chemokines and myeloperoxidase (MPO) activity (described below).

In another group of mice, knee joint hypernociception (described below) was evaluated and then knee joint samples (n = 5 mice/group) were collected for standard histological processing and Hematoxylin and Eosin (H&E) or toluidine blue (TB) staining. The H&E stained sections were scored for severity of synovial hyperplasia, intensity of the inflammatory infiltrate and bone erosion to obtain an arthritis index (range: 0–8) **[24].** TB-stained slides were used to estimate the joint proteoglycan content. The quantification of cartilage proteoglycan loss was conducted by evaluating the percentage of the TB-stained area in relation to the total evaluated cartilage surface **[24]** using the Image J software (National Institute of Health, Bethesda, MD, USA).

### Cytokine, chemokine, and MPO determination

Periarticular tissue was collected and homogenized in PBS containing antiproteases **[6].** Samples were processed and the supernatant was evaluated for concentrations of the chemokines CXCL1 and CXCL2 using commercially available ELISA assays, in accordance with the manufacturer’s instructions (R&D Systems, Minneapolis, MN). Culture cell supernatants (described below), were also assessed for the cytokines: interleukin IL-10, IL-17 and interferon-gamma IFN-γ using commercially available kits, according to manufacturer instructions (R&D Systems, Minneapolis, MN). Results were expressed as picograms of chemokines/cytokines (± S.E.M.) normalized for 100 mg tissue or 1 mL of culture supernatant.

Myeloperoxidase activity (a quantitative measurement of neutrophil sequestration) in periarticular tissue homogenates, standardized to the number of neutrophils obtained from the peritoneal cavity of casein-injected mice was assayed as described previously **[6].**

### Evaluation of hypernociception

The hypernociception of knee joints was measured as described earlier **[5].** Briefly, mice were placed in a quiet room in acrylic cages (12 × 10 × 17 cm in height) with a wire-grid floor for 15–30 minutes, before testing for environmental adaptation. Stimulations were performed only when mice were in quiet conditions. In these experiments, an electronic pressure meter (Insight Instruments model EFF-31, Ribeirão Preto, São Paulo, Brazil) consisting of a hand-held force transducer fitted with a large polypropylene tip (4.15 mm^2^) was used. Increasing perpendicular force was applied to the central area of the plantar surface of the hind paw to induce dorsal flexion of the tibiofemoral joint, followed by withdrawal of the paw. The electronic pressure meter automatically recorded the intensity of the force applied when the paw was withdrawn (in grams). The test was repeated until 3 measurements yielded consistent results (i.e., variation lower than 0.5 g). The hypernociception was tested before and after injection of saline or antigen, with results expressed as the change (Δ) in the withdrawal threshold. This was calculated by subtracting the zero-time mean measurements from the time-interval mean measurements.

### Intravital microscopy of the knee joint

Intravital microscopy was performed in the synovial microcirculation of the mouse knee, 24 h after antigen challenge, as described previously **[6].** Briefly, the patellar tendon was mobilized, partly resected so as the intraarticular synovial tissue of the left knee joint was then visualized (20-fold objective, 2–4 regions) for the determination of leukocyte rolling and adhesion. To measure the leukocyte–endothelial cell interactions, the fluorescent leukocyte marker rhodamine 6G (Sigma-Aldrich) was injected intravenously as a single bolus of 0.15 mg.kg^−1^ immediately before the measurements. Of note, these leukocytes are mostly neutrophils due to early stage analysis **[6]**. Rhodamine epiilumination was achieved with a 150W variable HBO mercury lamp in conjunction with a Zeiss filter set 15 (546/12-nm band-pass 2330 filter, 580-nm Fourier transforms, 590-nm late potentials; Zeiss, Wetzlar, Germany). The microscopic images were captured with a video camera (5100 HS; Panasonic, Secaucus, NJ) and recorded on DVD, using both filter blocks consecutively. Data analysis was performed off-line.

Rolling leucocytes were defined as those cells moving slower than the cells moving at a regular flux in a given vessel. The flux of rolling cells was measured as the number of rolling cells passing by a given point in the venule per minute, with results expressed as cells per minute. A leukocyte was considered to be adherent if it remained stationary for at least 30 seconds, and total leukocyte adhesion was quantified as the number of adherent cells within a 100 μm length of venule, with results expressed as cells/mm.

In another set of experiments, intravital microscopy was conducted in the knee joint of mice challenged with CXCL1 (dose 30 μg per knee, at 3 hours after challenge). This experiment aimed to investigate the potential anti-inflammatory effects of *L*. *muelleri*, CaCO_3_ or FR after direct injection of a chemotactic agent.

### Flow cytometry analysis

Popliteal lymph node cells were evaluated *ex vivo* for extracellular molecular expression patterns. Briefly, popliteal lymph nodes were removed from immunized mice, treated or not with *L*. *muelleri* (100 mg.kg^−1^, twice a day for 10 days), and 24 hours after antigen challenge, cells were isolated, and immediately stained for surface markers and then fixed with 2% formaldehyde. Preparations were then analyzed using a FACScan (Becton Dickinson USA), gating on a total lymphocytes, monocyte/macrophage and granulocyte populations. The antibodies used for the staining were rat immunoglobulin control(s), anti-CD4-FITC, anti-CD25-PE, anti-CD11c-FITC and anti-CD86-Alexa647 (all from Biolegend Inc, San Diego CA, USA). Popliteal lymph node cells were analyzed for their extracellular expression patterns and frequencies using the software Flow Jo 7.2 (Tree Star Inc, Ashland, USA). The frequency of positive cells was analyzed using a gate that included lymphocytes, large blast lymphocytes and monocytes/macrophages and granulocytes. Limits for the quadrant markers were always set based on negative populations and isotype controls.

### Splenocyte culture

Spleen of immunized mice, treated or not with *L*. *muelleri* (100 mg.kg^−1^, twice a day for 10 days), were collected 24 hours after antigen-challenge and splenocytes were isolated and then plated in 96-well microculture plates (1×10^6^ cells per well). Cells were re-stimulated with Concavalin-A (Con-A 2 μg.mL^−1^) or mBSA (100 μg.mL^−1^). Negative controls were stimulated with RPMI 1640 medium (Cultilab) only. Cells supernatants were harvested after 48 hours of stimulation for cytokine (IL-10, IL-17 and IFN-γ) measurements as described above.

### Statistical analysis

Data are presented as mean ± SEM and the statistical significance among control, AIA and treated groups was analyzed by analyses of variance (ANOVA), followed by Newman-Keuls *post hoc* analysis. Tests were performed with GraphPad Prism 4.0 software (GraphPad Software Inc., San Diego, CA, USA). Results with P<0.05 were considered statistically significant.

## Results

### *Lithothamnion muelleri* treatment inhibits articular inflammation in a dose-dependent manner

**Fig 1:**
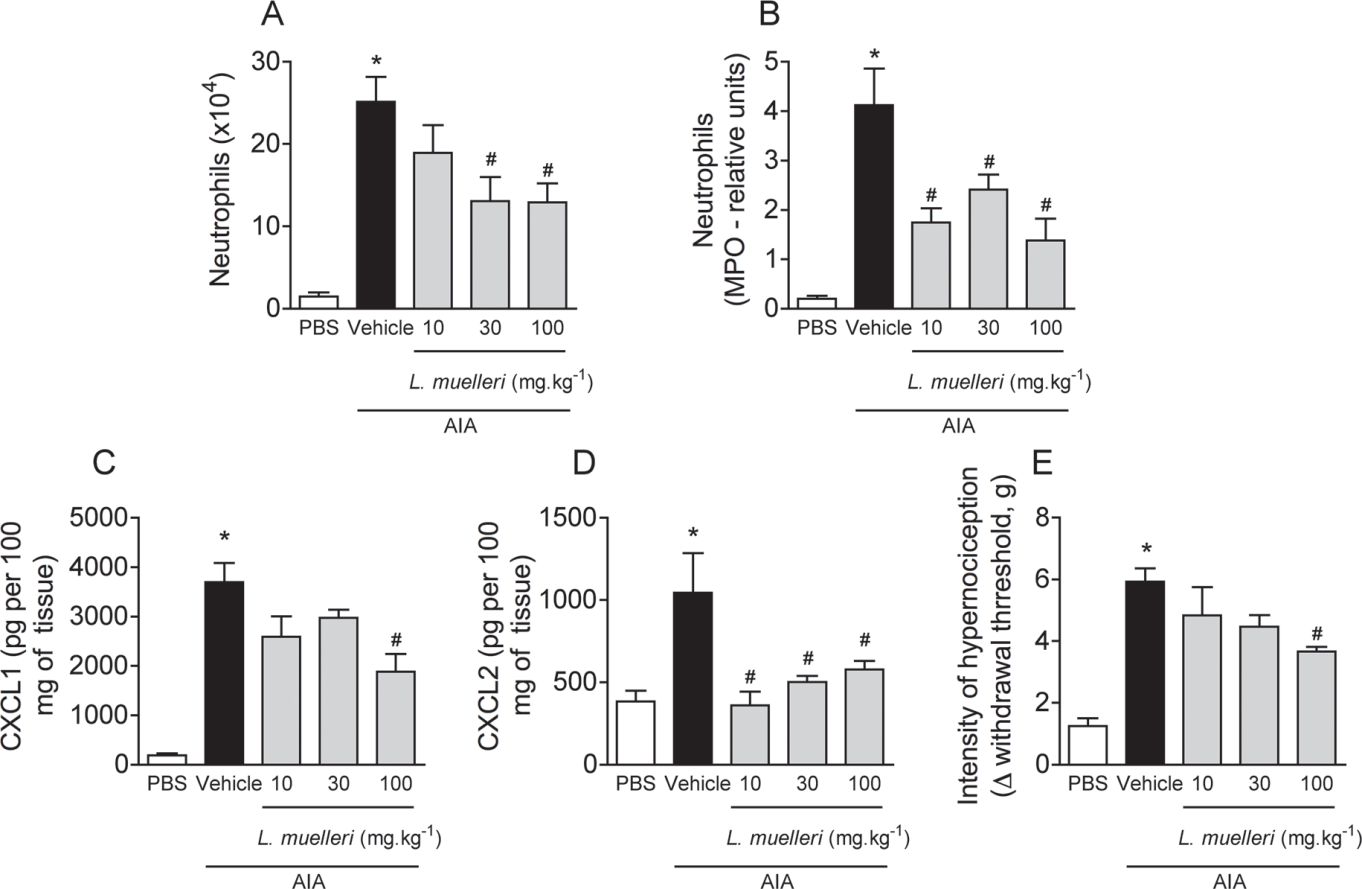
Effects of *L*.*muelleri* on AIA in mice. AIA mice were treated with vehicle (CMC 0.5% in filtered water) or with different doses (1, 10 or 100 mg.kg^−1^) of *L*. *muelleri*, orally, twice a day, during 10 days before antigen challenge in knee joint. The number of neutrophils in the synovial cavity (A), and the relative units of neutrophils in periarticular tissue, as determined by myeloperoxidase assay (B) were assessed 24 hours after 10 μg of mBSA or sterile saline (control) in knee joint of immunized mice. The concentrations of the chemokines CXCL1 (C) and CXCL2 (D) in the periarticular tissue were evaluated by ELISA. In E, hypernociception is presented as the change (Δ) in withdrawal threshold (in grams), calculated by subtracting the zero-time mean measurements from the time-interval mean measurements. The results are presented as the mean and SEM from 7 mice per group. * *P* <0.05 *versus* control mice; # for *P* < 0.05 *versus* vehicle-treated arthritic mice.

Immunized mice challenged with mBSA had a significant increase in neutrophil accumulation into the knee cavity (Fig. 1A) and periarticular tissue (Fig. 1B), and an increase in chemokine levels (CXCL1 and CXCL2) in periarticular tissue (Fig. 1C, D, respectively) when compared to vehicle-challenged mice. These inflammatory indices were reflected in articular dysfunction, once mBSA-challenged mice presented an increase of mechanical hypernociception (Fig. 1E). To verify the possible anti-inflammatory properties of *L*. *muelleri* in this model, a dose-response treatment (10, 30 or 100 mg.kg^−1^ BID, for 10 days before challenge) was performed. As observed in Fig. 1, the higher dose used (100 mg.kg^−1^) efficiently protected mice in all evaluated parameters, with reduction on neutrophil accumulation into the synovial cavity (Fig. 1A) and periarticular tissue (Fig. 1B), and reduction of CXCL1 (Fig. 1C) and CXCL2 (Fig. 1D) levels compared to vehicle-treated mice. In addition, the dose of 100 mg.kg^−1^ of *L*. *muelleri* was efficient in reducing the hypernociception index in arthritic mice (Fig. 1E).

In order to verify if a shorter period of treatment also interfered with the inflammatory response in this model, *L*. *muelleri* was given to immunized mice for five days before intra-articular challenge with mBSA. As seen in Table 1, all these parameters were reduced in 5 daily-treated mice compared to vehicle-treated mice, except for the hypernociception index (Table 1). Of note, treatment with *L*. *muelleri* for just one day before mBSA challenge did not have any effect on AIA (data not shown). As the maximum reduction of inflammatory parameters and hypernociception were obtained for *L*. *muelleri* at the dose of 100 mg.kg^−1^ during 10 days of treatment before challenge, all the subsequent experiments were performed using this dose and time point.

**Table 1:** Comparative analysis between different days of *L*.*muelleri* treatment on the reduction of inflammatory response in AIA.

Groups	Neutrophils (x 10^4^ in synovial cavity)	Neutrophils (Relative units)	CXCL1 (pg per 100 mg of tissue)	CXCL2 (pg per 100 mg of tissue)	Hypernociception (Δ withdrawal threshold, g)
PBS	1.50 ± 1.50	0.20 ± 0.05	220.9 ± 71.29	383.00 ± 121.1	1.24 ± 0.27
AIA + vehicle	23.70 ± 3.55*	3.92 ± 0.84*	2875.0 ± 549.9*	1371.0 ± 378.2*	6.41 ± 0.35*
AIA + *L*. *muelleri* (**5 d**)	8.97 ± 3.66^#^	1.92 ± 0.42^#^	1571.0 ± 201.7^#^	578.20 ± 50.74^#^	5.13 ± 0.39
AIA + *L*. *muelleri* (**10 d**)	8.07 ± 2.48^#^	0.43 ± 0.15^#^	1290.0 ± 150.8^#^	309.80 ± 106.6^#^	4.07 ± 0.56^#^

The treatments with L. muelleri (100mg/kg) were performed twice a day during 5 or 10 consecutive days. The results are presented as the mean and SEM from 7 mice per group.

* *P* <0.05 *versus* control mice;

# for *P* < 0.05 *versus* vehicle-treated arthritic mice.

### *Lithothamnion muelleri* treatment reduces cellular activation *in vivo* and *ex vivo*

**Fig 2:**
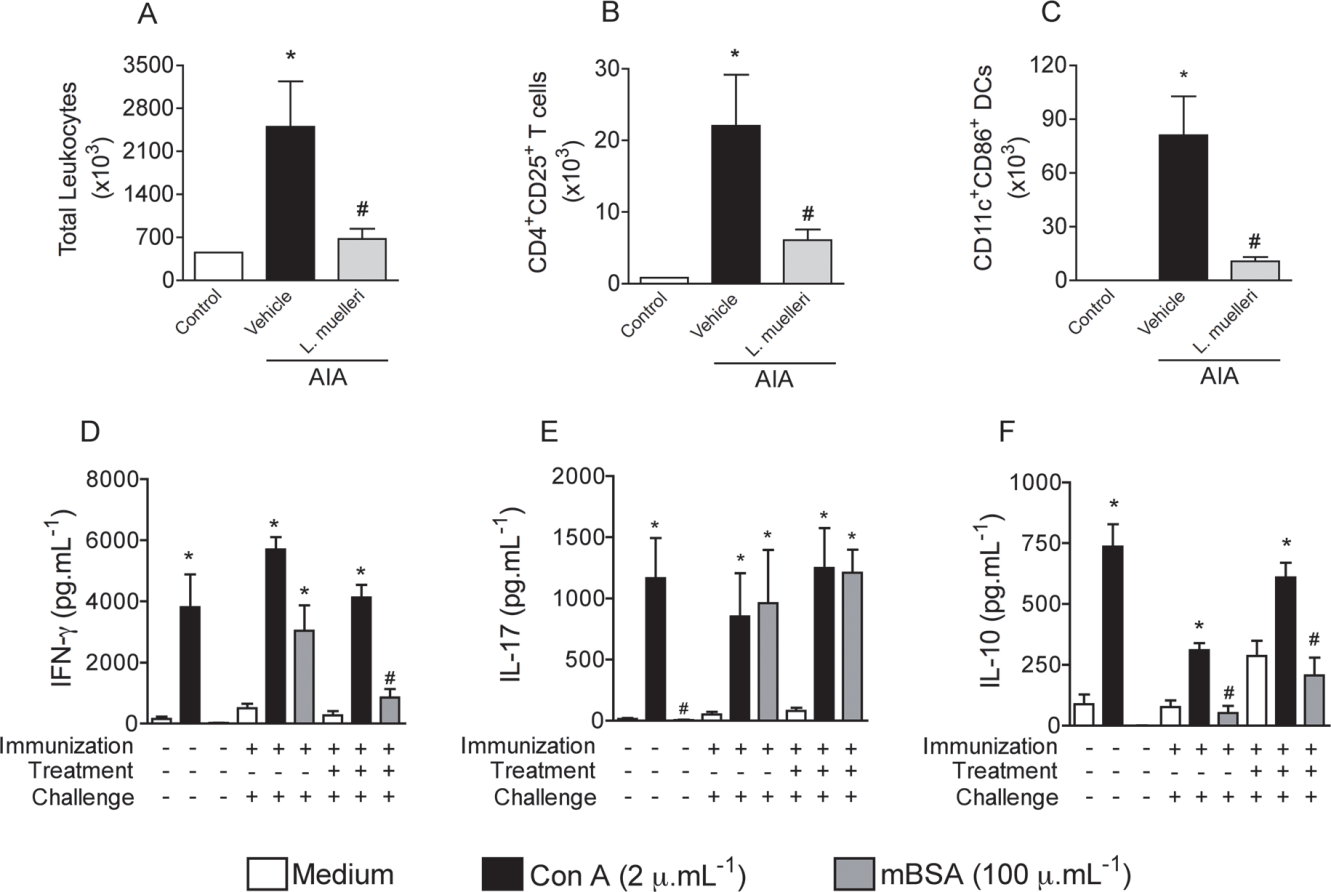
*In vivo* flow cytometry analyses from popliteal lymph node cells and *ex vivo* splenocyte antigen stimulation after *L*. *muelleri* treatment. AIA mice were treated as described before in Material and Methods. Twenty-four hours after joint challenge, the popliteal lymph node was collected and cells were isolated for quantifying the total cells numbers (Neubauer chamber) and assaying activated CD4 and DCs populations by cellular staining with labeled antibodies and FACS analysis. Results are expressed as numbers of total (A) or activated CD4^+^CD25^+^ (B), CD11c^+^CD86^+^ (C) cells in each population. In D-F, splenocytes were stimulated *ex vivo* with RPMI medium, 2 μg.mL^−1^ of Con-A or 100 μg.mL^−1^ of *L*. *muelleri* and culture supernatants were harvested 48 hours later for IFN-γ, IL-17 and IL-10 measurement by ELISA. Results are expressed as pg.mL^−1^ of culture supernatant. Bars show the mean and SEM results from 5 mice per group. * *P* <0.05 versus control mice; # for *P* < 0.05 versus vehicle-treated arthritic mice.

To test the efficacy of *L*. *muelleri* treatment in reducing cellular activation following mBSA challenge, popliteal lymph nodes and splenocytes from mBSA-challenged mice, previously treated or not with *L*. *muelleri*, and from PBS-challenged mice were harvested and analyzed by flow cytometry and ELISA assays. Popliteal lymph nodes removed after AIA showed increased number of total leucocytes (Fig. 2A), number of activated CD4^+^ CD25^+^ T cells (Fig. 2B) and activated CD11c^+^ CD86^+^ Dendritic cells—DCs (Fig. 2C) as compared to lymph nodes extracted from non-immunized and PBS-challenged mice. Treatment with *L*. *muelleri* markedly reduced number of total leucocytes (Fig. 2A) and number of activated cell types evaluated (Fig. 2B, C). Subsequently, we performed experiments to evaluate the ability of splenocytes isolated from immunized mice, treated or not with *L*. *muelleri*, to respond upon *ex-vivo* re-stimulation. After concanavalin (Con-A) stimulation, cells responded with intense production of IFN-γ (Fig. 2D), IL-17 (Fig. 2E) and IL-10 (Fig. 2F). Similarly, cells stimulated with mBSA produced the same effect, except for IL-10. In contrast, splenocytes obtained from mice previously treated with *L*. *muelleri* and re-stimulated *ex-vivo* with mBSA showed a marked reduction in the production of IFN-γ and IL-10, a pattern not seen after Con-A re-stimulation (Fig. 2D and 2F). However, there was no difference in the levels of IL-17 after mBSA stimulation of cells from the *L*. *muelleri* group (Fig. 2E). In order to test whether the algae treatment could interfere with the immunization process with mBSA, we quantified the amount of anti-mBSA antibodies in immunized mice. Fourteen days after immunization, both non-treated and *L*. *muelleri*-treated mice had the same amount of anti-mBSA (data not shown). Altogether, these *in vitro* data support the decreased activation of leucocytes observed in the knee joint *in vivo* and *ex vivo* (Fig. 2A-F) and suggest that *L*. *muelleri* treatment modulated the effector cellular responses upon the specific antigen stimulation without affecting the immunization process.

### The effects of *L*. *muelleri* are not due to the calcium carbonate present in its composition

*L*. *muelleri* has a high mineral content, majorly calcium carbonate (CaCO_3_), which accounts for 80–90% of its biomass **[25].** In this sense, the effects of CaCO_3_ on AIA were investigated and compared to the anti-inflammatory properties of *L*. *muelleri*.

**Fig 3:**
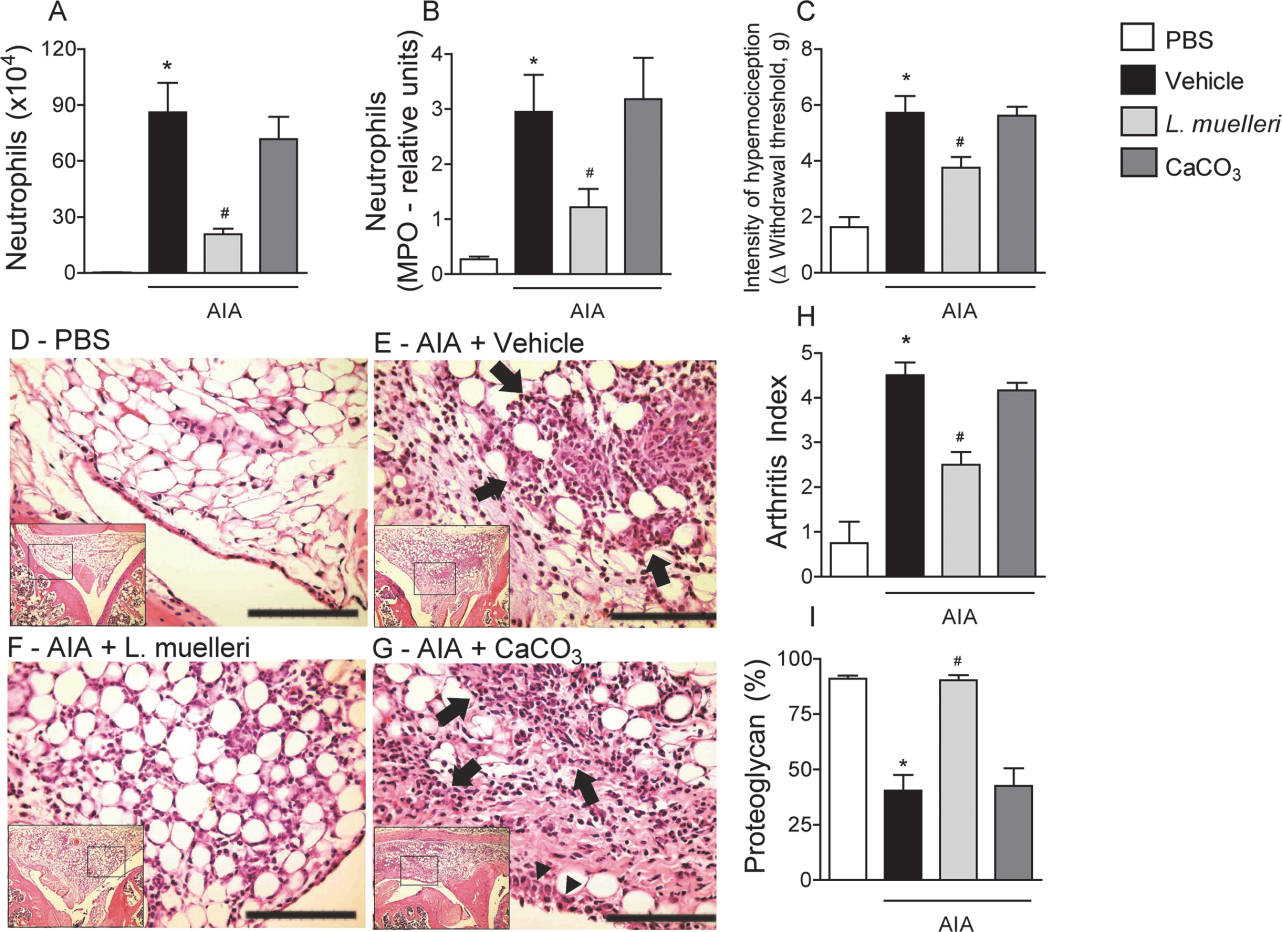
CaCO_3_ treatment of AIA mice has no anti-inflammatory and anti-nociceptive effects. AIA mice were treated as described in Material and Methods. The numbers of neutrophils in the synovial cavity (A), and the relative units of neutrophils in periarticular tissue, as determined by myeloperoxidase assay (B) were assessed 24 hours after injection of 10 μg mBSA or sterile saline (control) in knee joint of immunized mice. Hypernociception is presented as the change (Δ) in withdrawal threshold (in grams) (C). Representative H&E images of control (D), AIA + Vehicle (E), AIA + *L*. *muelleri* (F), AIA + CaCO_3_ (G) mice. AIA+ vehicle and AIA + CaCO_3_ groups (E and G, respectively) presented histopathological evidence of joint inflammation (inflammatory infiltrate [arrows], synovia hyperplasia [arrowheads], alteration of tissue architecture) compared with the other groups (D, F). Scale bars: 100 μm. Other parameters were evaluated as follows: (H), quantification of AIA arthritis index (described in Materials and Methods); and (I), quantification of proteoglycan loss, expressed in %. Results are presented as the mean and SEM results from 5 mice per group. * *P* <0.05 versus control mice; # for *P* < 0.05 versus vehicle-treated arthritic mice.

In contrast to the treatment with *L*. *muelleri*, the treatment with CaCO_3_ did not promote any improvement of inflammatory parameters on arthritic mice, as demonstrated by neutrophil recruitment (Fig. 3A, B) or in the hypernociception index (Fig. 3C) when compared to vehicle-treated mice.

Quantification of the histological features concurred with the qualitative aspects found in the synovial tissue. Challenge with mBSA led to intense joint inflammation, as demonstrated by infiltration of polymorphonuclear cells into the synovium and periarticular tissues (Fig. 3A and 3E) as well as by synovial hyperplasia (not shown). In addition, the arthritic index (Fig. 3H) and loss of proteoglycan in joint cartilage (Fig. 3I) were substantially increased in vehicle-treated mice compared to non-arthritic mice. The treatment with *L*. *muelleri* significantly decreased joint inflammation (Fig. 3F), arthritic index (Fig. 3H) and proteoglycan loss in joint cartilage (Fig. 3I), while the treatment with CaCO_3_ did not modify significantly any of these parameters (Fig. 3G-I).

### Polysaccharide-rich fraction from *L*. *muelleri* exhibit anti-inflammatory and anti-hypernociceptive properties during AIA in mice

**Fig 4:**
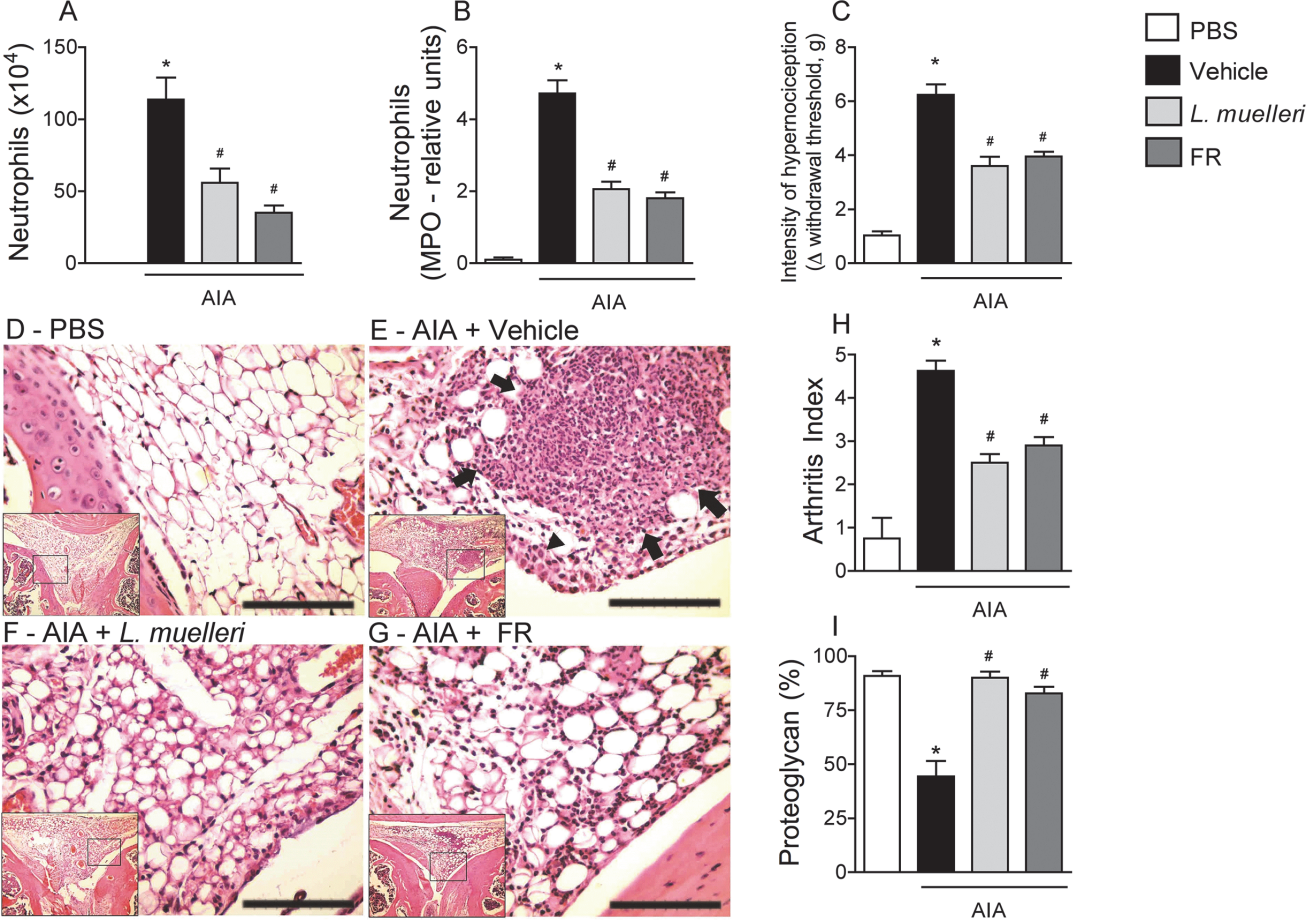
Sulfated polysaccharides from *L*. *muelleri* present anti-inflammatory and anti-nociceptive properties in AIA. AIA mice were treated with vehicle (CMC 0.5% in filtered water), *L*. *muelleri* (100 mg.kg^−1^) or FR (1 mg.kg^−1^), orally, twice a day, during 10 days before antigen challenge in knee joint. The numbers of neutrophils in the synovial cavity (A), and the relative units of neutrophils in periarticular tissue, as determined by myeloperoxidase assay (B) were assessed 24h after joint challenge. Hypernociception is presented as the change (Δ) in withdrawal threshold (in grams) (C). Representative H&E images of control (D), AIA + vehicle (E), AIA + *L*. *muelleri* (F), AIA + FR (G) mice. AIA + vehicle group (E) presented histopathological evidence of joint inflammation (inflammatory infiltrate [arrows], synovial hyperplasia [arrowheads], alteration of tissue architecture) compared with the other groups (D, F and G). Scale bars: 100 μm. Other parameters were evaluated as follows: (H), quantification of AIA arthritis index (described in Materials and Methods); and (I), quantification of proteoglycan loss, expressed in %. Bars show the mean and SEM results from 6 mice per group. * *P* <0.05 versus control mice; # for *P* < 0.05 versus vehicle-treated arthritic mice.

Sulfated polysaccharides from seaweeds are known to possess biological activities such as anticoagulant, antioxidant, antitumor, antiviral and anti-inflammatory **[26].** Therefore, the role of a fraction enriched in sulfated polysaccharides (FR) derived from *L*. *muelleri* was investigated in this study. Similarly to *L*. *muelleri*, the pre-treatment with FR (1 mg.kg) reduced the inflammatory response after mBSA challenge, with a significantly decrease of neutrophil accumulation into the joint cavity (Fig. 4A) and periarticular tissue (Fig. 4B), as well as in reduction of the hypernociceptive threshold (Fig. 4C). Histological analysis of knee joint revealed that the treatment with FR promoted reduced cellular recruitment to joint after mBSA challenge (Fig. 4G), following reduced arthritis index (Fig. 4H) and loss of proteoglycan (Fig. 4I) in comparison to vehicle-treated mice. Of note, all the reduced inflammatory parameters observed in FR-treated group were similar to *L*. *muelleri* treatment (Fig. 4F, 4H, I). Taken in account the negative results obtained with CaCO_3_ treatment, the results herein reported indicate that the anti-inflammatory and anti-nociceptive effects of *L*. *muelleri* are due to the presence of sulfated polysaccharides in its composition.

### *L*. *muelleri* treatment inhibits leukocyte-endothelial cell interactions on the joint microvasculature

**Fig 5:**
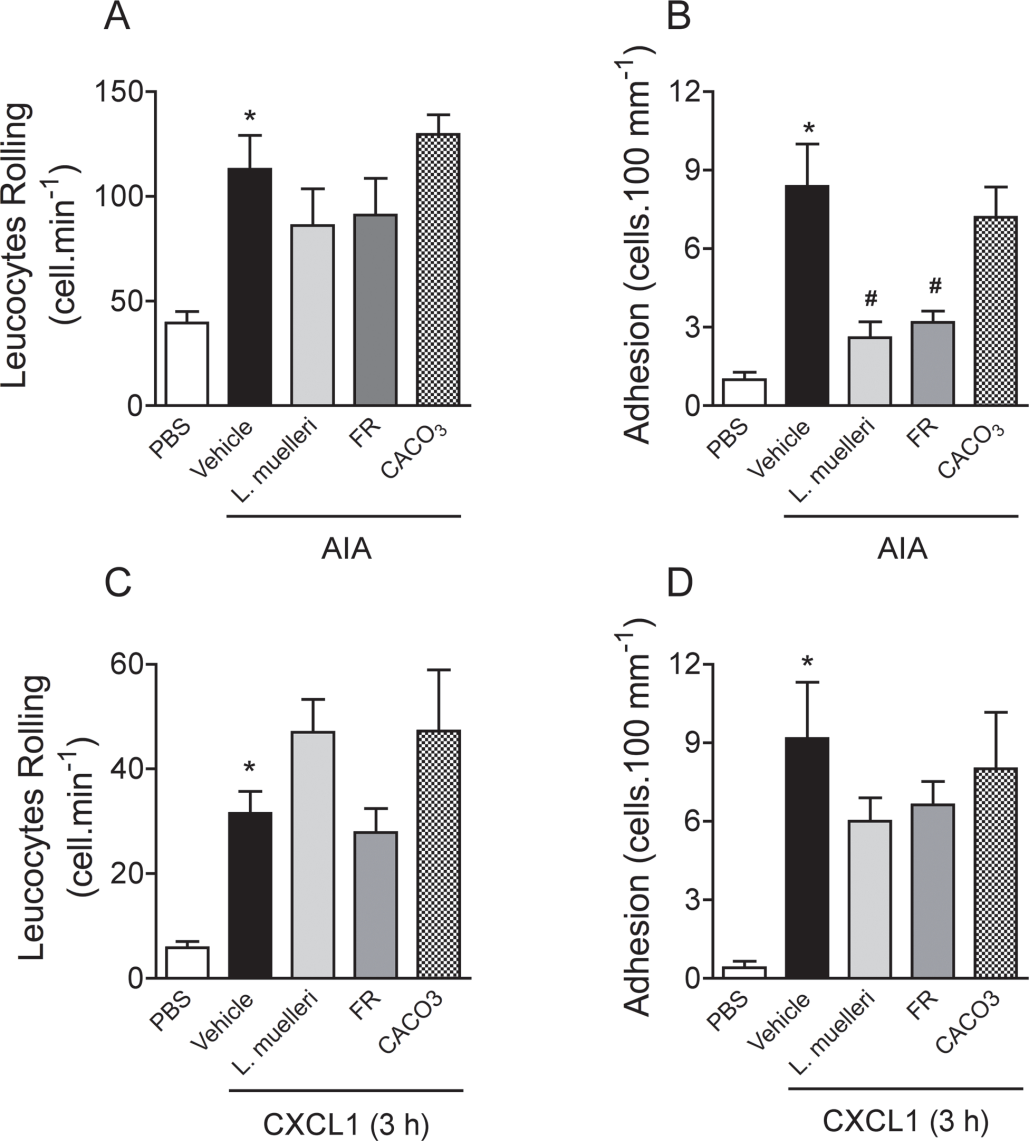
Effects of *L*. *muelleri* and FR on the interaction between leucocytes and endothelial cells in the synovial microvasculature. AIA mice were treated with vehicle solution (CMC 0.5% in filtered water), *L*. *muelleri* (100 mg.kg^−1^), CaCO_3_ (100 mg.kg^−1^) or FR (1 mg.kg^−1^) orally, twice a day, during 10 days before antigen challenge in knee joint or before CXCL1 (30 μg per knee) challenge in non-immunized mice. Rolling (A-C) and adhesion (B-D) of leucocytes to the synovial endothelium were assessed 24 hours after joint challenge with mBSA (A-B) or 3 hours after CXCL1 (30 μg per knee) knee challenge (C-D). The flux of rolling cells was measured as the number of rolling cells passing by a given point in the venule per minute. A leukocyte was considered to be adherent if it remained stationary for at least 30 seconds, and total leukocyte adhesion was quantified as the number of adherent cells within a 100-μm length of venule. Bars show the mean and SEM results from 5 mice per group. * *P* <0.05 versus control mice; # for *P* < 0.05 versus vehicle-treated arthritic mice.

As shown above, the treatment of AIA mice with *L*. *muelleri* reduced the joint production of the neutrophil-related chemokines CXCL1 and CXCL2 (Fig. 1C and 1D, respectively). Such inhibition could account for the local impairment of neutrophil accumulation into periarticular tissues (Fig. 1B). In this regard, a series of experiments were conducted to evaluate whether the effects of *L*. *muelleri* or FR in AIA correlated with the ability to prevent interactions between leucocytes and synovial microvessels, using intravital microscopy **[6].** Intra-articular antigen challenge in immunized mice treated with vehicle was accompanied by an increase in leukocyte rolling and adhesion (Fig. 5A and 5B, respectively). As seen in Fig. 5A, the treatment of mice with *L*. *muelleri* or FR had no effect in reducing leukocyte rolling (Fig. 5A). However, the *L*. *muelleri* and FR treatments reduced about 65% of leukocyte adhesion (Fig. 5B) to the synovial microvessels. Conversely, the CaCO_3_ treatment had no effect in any of these parameters (Fig. 5B).

Since chemokines are directly involved in leukocyte recruitment during inflammation and the data showed that *L*. *muelleri* treatment reduced CXCL1 and CXCL2 production (Fig. 1C and 1D, respectively), the direct effects of *L*. *muelleri* on leukocyte rolling and adhesion after CXCL1 challenge was investigated. Mice previously treated with vehicle solution, CaCO_3,_ *L*. *muelleri* or FR were challenged with the CXCL1 chemokine into knee joint and intravital microscopy was conducted 3 hours later. Injection of CXCL1 chemokine induced the rolling and adhesion of leucocytes into synovial microvessels of vehicle-treated mice (Fig. 5C, D, respectively). However, as seen in Fig. 5 (C, D), none of the treatments were able to reduce the rolling or adherence of cells induced by CXCL1 knee challenge. Therefore, the data depicted here indicate that the treatment with *L*. *muelleri* or its bioactive rich sulfated polysaccharides impaired leukocyte adhesion to the synovial microvessels by decreasing CXCL1/CXCL2 levels during the prophylactic treatment.

## Discussion and Conclusions

In the present study, we highlighted the anti-inflammatory and anti-hypernociceptive effects of the treatment with the red marine algae *Lithothamnion muelleri* in an experimental model of arthritis (AIA) in mice. The major results of the present study can be summarized as follow: 1) treatment of AIA mice with *L*. *muelleri* reduced inflammation in a time and dose-dependent manner, as demonstrated by the reduction in numbers and activation of leucocytes in draining lymph nodes and knee joint, as well as a reduction in the levels of the chemokines CXCL1 and CXCL2; 2) *L*. *muelleri* treatment markedly reduced tissue damage and hypernociception after AIA induction; 3) *L*. *muelleri* treatment impaired leukocyte recruitment in synovial microvessels of knee; 4) The anti-inflammatory and anti-hypernociceptive effects of *L*. *muelleri* treatment may be attributed to the sulfated polysaccharides present in its composition.

Preparations containing *Lithothamnion* spp. is commercialized as food supplements in different countries, as sources of calcium and other mineral components. The chemical composition of red alga also comprises large amounts of sulfated water-soluble polysaccharides, to which several anti-inflammatory properties were attributed **[18–19, 22, 27].** The biological effects elicited by poly- and oligosaccharides depend on the nature of the constitutive monosaccharides as well as the stereochemistry of the linkages **[28].** We demonstrated herein that the treatment with the crude extract of *L*. *muelleri* or its polysaccharide-rich fraction (FR) was able to marked reduce the total number of leucocytes, especially neutrophils, into the synovial cavity and periarticular tissue of mice subjected to AIA.

Neutrophils are one of the most abundant cells present in the affected joints of RA patients, including synovial fluid and panus/cartilage interface **[29–31].** These cells are important sources of proinflammatory mediators, including cytokines and chemokines, reactive oxygen species-producing enzymes and proteases, present critical role in initiating and maintaining the inflammatory process in arthritic joints **[4, 6, 32].** Accordingly, we have previous demonstrated that the blockade of CXCR1/CXCR2 receptors with two different allosteric inhibitors, reparixin or DF2162, decreased neutrophil recruitment, an effect that was associated with marked inhibition of neutrophil adhesion into the synovial microcirculation of knee of mice **[6,33].** Here, the levels of the neutrophil-related chemokines CXCL1 and CXCL2 were reduced in *L*. *muelleri* treated mice, preventing neutrophil accumulation into knee joints and tissue damage of AIA mice. However, this treatment was not effective in reducing the pro-inflammatory cytokines IFN-γ and TNF-α in the knee joint when compared to vehicle-treated AIA mice (data not shown).

The process of leukocyte extravasation from the blood into the inflamed tissues requires a complex cascade of events between the leucocytes and the endothelium, including leukocyte rolling, adhesion, crawling and migration **[34].** Using intravital microscopy we demonstrated that treatment with *L*. *muelleri* or its polysaccharide-rich fraction suppressed the firm adhesion step of leukocyte interaction to endothelial cells. Interestingly, we demonstrated that these effects might be due to reduction of CXCL1 and CXCL2 levels in *L*. *muelleri* AIA treated mice, since the treatment with *L*. *muelleri* of non-immunized mice did not prevent leukocyte adhesion induced by locally injection of the chemokine CXCL1. Similarly, Matsui *et al*. (2003) demonstrated that polysaccharides from the red microalgae *Porphyridium* primary inhibited the migration of PMNs toward formylated peptides, and also partially blocked adhesion of PMNs to endothelial cells **[19].** Indeed, the antiadhesive activity of polyssacharide-rich fractions from *L*. *muelleri* on cremaster tissue after lipopolysaccharide from *Escherichia coli* stimulus using intravital microscopy was also demonstrated **[18].** In this study, the intravenous injection of the polysaccharide-rich fractions reduced leukocyte rolling and adherence (data not shown) in the same manner as found in fucoidan treated mice **[18].** Finally, we also demonstrated that *L*. *muelleri* treatment controls inflammatory responses, tissue injury and mortality associated with Graft-*versus*-Host disease via inhibition of leukocyte interactions with intestinal venules **[22].** Altogether, we suggest here that the amelioration of arthritis in *L*. *muelleri*-treated mice seems to be, at least in part, secondary to reduction neutrophil recruitment and CXCL1 and CXCL2 release, in a way dependent on inhibition of neutrophil adhesion into the synovial microcirculation of knee. However, further studies need to be conducted to clarify the exact mechanism(s) by which *L*.*muelleri* exerts its anti-inflammatory and analgesic effects.

Joint pain are a markedly symptom in RA patients and also can be investigated in arthritic experimental models **[6, 33].** Here, mice treated with *L*. *muelleri* presented markedly reduction in joint inflammation and inflammatory hypernociception after AIA induction. Supporting these findings, two clinical trials studies in humans supplemented with a multi-mineral supplement (Aquamin F) prepared from *Lithothamnion corallioides* demonstrated a relief of osteoarthritis symptoms, described as a reduction in pain and stiffness, as well as by an improvement of daily activities of subjects diagnosed with moderate to severe osteoarthritis of the knee **[15–16].** In addition, several studies have reported the anti-inflammatory and analgesic effects of dietary supplements containing polysaccharides derived from marine algae in different systems **[27, 35–38].** Also, directly associated with our studies, several papers have described the essential role of neutrophils in the induction of inflammatory hypernociception induced by different stimuli **[6, 33, 39–40].** Furthermore, Figueiredo *et al*. (2010) demonstrated that a lectin isolated from the red marine algae *Hypnea cervicornis* inhibited inflammatory hypernociception, being the effect was associated to preventing neutrophil recruitment **[41].**

In addition to neutrophils, other cellular types are also important components for pathogenesis of RA, including dendritic and T cells, which contribute to pannus formation and cytokine release (as reviewed in **[42]).** Here, we demonstrated that treatment with *L*. *muelleri* caused marked reduction of activated CD4^+^ CD25^+^ T cells and CD11c^+^ CD86^+^ DCs in draining popliteal lymph nodes of mBSA challenged mice. In addition, mBSA re-stimulation of splenocytes of immunized-treated mice resulted in reduction of IFN-γ and IL-10 levels, without altering IL-17 levels or anti-mBSA antibodies. These data suggest that *L*. *muelleri* treatment do not alter the immunization process and also reveal possible immunomodulatory properties for the constituent sulfated polysaccharides, which deserve further investigation.

In conclusion, we have demonstrated that the treatment of *L*. *muelleri* crude extract as well as its polysaccharide-rich fraction was effective to control the articular inflammatory response and the intensity of hypernociception in an mBSA model of arthritis. *L*. *muelleri* treatment reduced neutrophil migration to site of inflammation as well as the production of chemokines in a way dependent on leukocyte adhesion to the endothelium. Altogether, these results point out *L*. *muelleri* as promising source of bioactive polysaccharides for treating the articular disorders-associated inflammation.

## References

[1] McInnes IB, Schett G (2007) Cytokines in the pathogenesis of rheumatoid arthritis. Nat Rev Immunol 7: 429–442. 17525752 10.1038/nri2094

[2] McInnes IB, Schett G (2011) The pathogenesis of rheumatoid arthritis. N Engl J Med 365: 2205–2219. 10.1056/NEJMra1004965 22150039

[3] Kitsis E, Weissmann G (1991) The role of the neutrophil in rheumatoid arthritis. Clin Orthop Relat Res: 63–72. 2009678

[4] Wipke BT, Allen PM (2001) Essential role of neutrophils in the initiation and progression of a murine model of rheumatoid arthritis. J Immunol 167: 1601–1608. 11466382 10.4049/jimmunol.167.3.1601

[5] Sachs D, Coelho FM, Costa VV, Lopes F, Pinho V, et al (2011) Cooperative role of tumour necrosis factor-alpha, interleukin-1beta and neutrophils in a novel behavioural model that concomitantly demonstrates articular inflammation and hypernociception in mice. Br J Pharmacol 162: 72–83. 10.1111/j.1476-5381.2010.00895.x 20942867 PMC3012407

[6] Coelho FM, Pinho V, Amaral FA, Sachs D, Costa VV, et al (2008) The chemokine receptors CXCR1/CXCR2 modulate antigen-induced arthritis by regulating adhesion of neutrophils to the synovial microvasculature. Arthritis Rheum 58: 2329–2337. 10.1002/art.23622 18668539

[7] Grespan R, Fukada SY, Lemos HP, Vieira SM, Napimoga MH, et al (2008) CXCR2-specific chemokines mediate leukotriene B4-dependent recruitment of neutrophils to inflamed joints in mice with antigen-induced arthritis. Arthritis Rheum 58: 2030–2040. 10.1002/art.23597 18576322

[8] Wright HL, Moots RJ, Bucknall RC, Edwards SW (2010) Neutrophil function in inflammation and inflammatory diseases. Rheumatology (Oxford) 49: 1618–1631. 10.1093/rheumatology/keq045 20338884

[9] Smolen JS, Aletaha D, Koeller M, Weisman MH, Emery P (2007) New therapies for treatment of rheumatoid arthritis. Lancet 370: 1861–1874. 17570481 10.1016/S0140-6736(07)60784-3

[10] Weinblatt ME, Kremer JM, Bankhurst AD, Bulpitt KJ, Fleischmann RM, et al (1999) A trial of etanercept, a recombinant tumor necrosis factor receptor:Fc fusion protein, in patients with rheumatoid arthritis receiving methotrexate. N Engl J Med 340: 253–259 9920948 10.1056/NEJM199901283400401

[11] Lipsky PE, van der Heijde DM, St Clair EW, Furst DE, Breedveld FC, et al (2000) Infliximab and methotrexate in the treatment of rheumatoid arthritis. Anti-Tumor Necrosis Factor Trial in Rheumatoid Arthritis with Concomitant Therapy Study Group. N Engl J Med 343: 1594–1602. 11096166 10.1056/NEJM200011303432202

[12] National Center for Complementary and Integrative Health (NIH) website. Available: http://nccam.nih.gov/about/plans/2011. Accessed 12 Jul 2014.

[13] Boisset M, Fitzcharles MA (1994) Alternative medicine use by rheumatology patients in a universal health care setting. J Rheumatol 21: 148–152. 8151571

[14] Rao JK, Mihaliak K, Kroenke K, Bradley J, Tierney WM, et al (1999) Use of complementary therapies for arthritis among patients of rheumatologists. Ann Intern Med 131: 409–416. 10498556 10.7326/0003-4819-131-6-199909210-00003

[15] Frestedt JL, Walsh M, Kuskowski MA, Zenk JL (2008) A natural mineral supplement provides relief from knee osteoarthritis symptoms: a randomized controlled pilot trial. Nutr J 7: 9 10.1186/1475-2891-7-9 18279523 PMC2265739

[16] Frestedt JL, Kuskowski MA, Zenk JL (2009) A natural seaweed derived mineral supplement (Aquamin F) for knee osteoarthritis: a randomised, placebo controlled pilot study. Nutr J 8: 7 10.1186/1475-2891-8-7 19187557 PMC2642861

[17] Faulkner DJ (2000) Marine natural products. Nat Prod Rep 17: 7–55. 10714898 10.1039/a809395d

[18] Soares CM, Malagoli BG, Menezes, Pinho V, Souza DG, et al (2012) Antiadhesive activity of polysaccharide-rich fractions from Lithothamnion muelleri. Z Naturforsch C 67: 391–397. 23016278 10.1515/znc-2012-7-806

[19] Matsui MS, Muizzuddin N, Arad S, Marenus K (2003) Sulfated polysaccharides from red microalgae have antiinflammatory properties in vitro and in vivo. Appl Biochem Biotechnol 104: 13–22. 12495202 10.1385/abab:104:1:13

[20] Aslam MN, Paruchuri T, Bhagavathula N, Varani J (2010) A mineral-rich red algae extract inhibits polyp formation and inflammation in the gastrointestinal tract of mice on a high-fat diet. Integr Cancer Ther 9: 93–99. 10.1177/1534735409360360 20150219 PMC2861409

[21] Malagoli BG, Cardozo FTGS, Gomes JHS, Ferraz VP, Simoes CMO, et al (2014) Chemical characterization and antiherpes activity of sulfated polysaccharides from Lithothamnion muelleri. Int. Journal of Biological Macromolecules. 66:332–337. 10.1016/j.ijbiomac.2014.02.053 24608026

[22] Rezende BM, Bernardes PTT, Resende CB, Arantes RME, Souza DG et al (2013) Lithothamnion muelleri controls inflammatory responses, target organ injury and lethality associated with graft-versus-host disease in mice. Mar. Drugs. 11:2595–2615. 10.3390/md11072595 23873335 PMC3736440

[23] Zimmermann M (1983) Ethical guidelines for investigations of experimental pain in conscious animals. Pain 16: 109–110. 6877845 10.1016/0304-3959(83)90201-4

[24] Queiroz-Junior CM, Madeira MF, Coelho FM, Costa VV, Bessoni RL, et al (2011) Experimental arthritis triggers periodontal disease in mice: involvement of TNF-alpha and the oral Microbiota. J Immunol 187: 3821–3830. 10.4049/jimmunol.1101195 21890656

[25] Dias GTM (2000) Granulados bioclásticos—Algas calcárias. Revista Brasileira de Geofísica 18: 307–318.

[26] Jiao G, Yu G, Zhang J, Ewart HS (2011) Chemical structures and bioactivities of sulfated polysaccharides from marine algae. Mar Drugs 9: 196–223. 10.3390/md9020196 21566795 PMC3093253

[27] Chaves Lde S, Nicolau LA, Silva RO, Barros FC, Freitas AL, et al (2013) Antiinflammatory and antinociceptive effects in mice of a sulfated polysaccharide fraction extracted from the marine red algae Gracilaria caudata. Immunopharmacol Immunotoxicol 35: 93–100. 10.3109/08923973.2012.707211 22830978

[28] Courtois J (2009) Oligosaccharides from land plants and algae: production and applications in therapeutics and biotechnology. Curr Opin Microbiol 12: 261–273 10.1016/j.mib.2009.04.007 19467920

[29] Bender JG, Van Epps DE, Searles R, Williams RC Jr (1986) Altered function of synovial fluid granulocytes in patients with acute inflammatory arthritis: evidence for activation of neutrophils and its mediation by a factor present in synovial fluid. Inflammation 10: 443–453. 3025094 10.1007/BF00915828

[30] Edwards SW, Hughes V, Barlow J, Bucknall R (1988) Immunological detection of myeloperoxidase in synovial fluid from patients with rheumatoid arthritis. Biochem J 250: 81–85 2833238 10.1042/bj2500081 PMC1148818

[31] Kowanko IC, Ferrante A (1996) Adhesion and TNF priming in neutrophil-mediated cartilage damage. Clin Immunol Immunopathol 79: 36–42. 8612349 10.1006/clin.1996.0048

[32] Tanaka D, Kagari T, Doi H, Shimozato T (2006) Essential role of neutrophils in anti-type II collagen antibody and lipopolysaccharide-induced arthritis. Immunology 119: 195–202. 16836650 10.1111/j.1365-2567.2006.02424.x PMC1782359

[33] Amaral FA, Costa VV, Tavares LD, Sachs D, Coelho FM, et al (2012) NLRP3 inflammasome-mediated neutrophil recruitment and hypernociception depend on leukotriene B(4) in a murine model of gout. Arthritis Rheum 64: 474–484. 10.1002/art.33355 21952942

[34] Kolaczkowska E, Kubes P (2013) Neutrophil recruitment and function in health and inflammation. Nat Rev Immunol 13: 159–175. 10.1038/nri3399 23435331

[35] Guzman S, Gato A, Calleja JM (2001) Antiinflammatory, analgesic and free radical scavenging activities of the marine microalgae Chlorella stigmatophora and Phaeodactylum tricornutum. Phytother Res 15: 224–230. 11351357 10.1002/ptr.715

[36] Guzman S, Gato A, Lamela M, Freire-Garabal M, Calleja JM (2003) Anti-inflammatory and immunomodulatory activities of polysaccharide from Chlorella stigmatophora and Phaeodactylum tricornutum. Phytother Res 17: 665–670. 12820237 10.1002/ptr.1227

[37] Assreuy AM, Gomes DM, da Silva MS, Torres VM, Siqueira RC, et al (2008) Biological effects of a sulfated-polysaccharide isolated from the marine red algae Champia feldmannii. Biol Pharm Bull 31: 691–695. 18379064 10.1248/bpb.31.691

[38] Rodrigues JA, Vanderlei ES, Silva LM, Araujo IW, Queiroz IN, et al (2012) Antinociceptive and anti-inflammatory activities of a sulfated polysaccharide isolated from the green seaweed Caulerpa cupressoides. Pharmacol Rep 64: 282–292. 22661177 10.1016/s1734-1140(12)70766-1

[39] Levine JD, Lau W, Kwiat G, Goetzl EJ (1984) Leukotriene B4 produces hyperalgesia that is dependent on polymorphonuclear leukocytes. Science 225: 743–745. 6087456 10.1126/science.6087456

[40] Cunha TM, Verri WA Jr, Schivo IR, Napimoga MH, Parada CA, et al (2008) Crucial role of neutrophils in the development of mechanical inflammatory hypernociception. J Leukoc Biol 83: 824–832. 10.1189/jlb.0907654 18203872

[41] Figueiredo JG, Bitencourt FS, Cunha TM, Luz PB, Nascimento KS, et al (2010) Agglutinin isolated from the red marine alga Hypnea cervicornis J. Agardh reduces inflammatory hypernociception: involvement of nitric oxide. Pharmacol Biochem Behav 96: 371–377 10.1016/j.pbb.2010.06.008 20600247

[42] Klareskog L, Catrina AI, Paget S (2009) Rheumatoid arthritis. Lancet 373: 659–672. 10.1016/S0140-6736(09)60008-8 19157532

